# A study of ticks and tick-borne livestock pathogens in Pakistan

**DOI:** 10.1371/journal.pntd.0005681

**Published:** 2017-06-26

**Authors:** Shahid Karim, Khemraj Budachetri, Nabanita Mukherjee, Jaclyn Williams, Asma Kausar, Muhammad Jawadul Hassan, Steven Adamson, Scot E. Dowd, Dmitry Apanskevich, Abdullah Arijo, Zia Uddin Sindhu, Muhammad Azam Kakar, Raja Muhammad Dilpazir Khan, Shafiq Ullah, Muhammad Sohail Sajid, Abid Ali, Zafar Iqbal

**Affiliations:** 1 Department of Biological Sciences, University of Southern Mississippi, Hattiesburg, MS, United States of America; 2 Department of Parasitology, University of Agriculture, Faisalabad, Pakistan; 3 Molecular Research LP, Shallowater, TX, United States of America; 4 Institute of Arthropodology and Parasitology, Georgia Southern University, Statesboro, Georgia, United States of America; 5 Department of Parasitology, Sindh Agricultural University, Tando Jam, Pakistan; 6 Faculty of Veterinary and Animal Sciences, Lasbela University of Agriculture, Water and Marine Sciences, Lasbela, Balochistan, Pakistan; 7 Department of Animal Husbandry, Muzaffarabad, Azad Jammu and Kashmir, Pakistan; 8 Department of Zoology, Abdul Wali Khan University, Mardan, Pakistan; The Kenya Medical Research Institute (KEMRI), KENYA

## Abstract

**Background:**

As obligate blood-feeding arthropods, ticks transmit pathogens to humans and domestic animals more often than other arthropod vectors. Livestock farming plays a vital role in the rural economy of Pakistan, and tick infestation causes serious problems with it. However, research on tick species diversity and tick-borne pathogens has rarely been conducted in Pakistan. In this study, a systematic investigation of the tick species infesting livestock in different ecological regions of Pakistan was conducted to determine the microbiome and pathobiome diversity in the indigenous ticks.

**Methodology/Principal findings:**

A total of 3,866 tick specimens were morphologically identified as 19 different tick species representing three important hard ticks, *Rhipicephalus*, *Haemaphysalis* and *Hyalomma*, and two soft ticks, *Ornithodorus* and *Argas*. The bacterial diversity across these tick species was assessed by bacterial *16S rRNA* gene sequencing using a 454-sequencing platform on 10 of the different tick species infesting livestock. The notable genera detected include *Ralstonia*, *Clostridium*, *Staphylococcus*, *Rickettsia*, *Lactococcus*, *Lactobacillus*, *Corynebacterium*, *Enterobacter*, and *Enterococcus*. A survey of Spotted fever group rickettsia from 514 samples from the 13 different tick species generated rickettsial-specific amplicons in 10% (54) of total ticks tested. Only three tick species *Rhipicephalus microplus*, *Hyalomma anatolicum*, and *H*. *dromedarii* had evidence of infection with “*Candidatus* Rickettsia amblyommii” a result further verified using a *rompB* gene-specific quantitative PCR (qPCR) assay. The *Hyalomma* ticks also tested positive for the piroplasm, *Theileria annulata*, using a qPCR assay.

**Conclusions/Significance:**

This study provides information about tick diversity in Pakistan, and pathogenic bacteria in different tick species. Our results showed evidence for *Candidatus* R. amblyommii infection in *Rhipicephalus microplus*, *H*. *anatolicum*, and *H*. *dromedarii* ticks, which also carried *T*. *annulata*.

## Author summary

Ticks are known for their negative impact on animal and human health through infestation and are capable of transmitting a wide range of pathogens including protozoan, viruses, and bacteria such as the spirochetes and rickettsiae. Ticks are widely distributed in different ecological, and geographical regions of Pakistan. Tick-borne diseases, such as bovine babesiosis and theileriosis have been reported in Pakistan. Crimean-Congo hemorrhagic fever (CCHF), a tick-borne viral disease in Pakistan, affects individuals who have close contact with livestock, including those working in slaughterhouses, veterinary practices, and hospitals. In Pakistan, CCHF is vectored by *Hyalomma* tick species, and therefore a significant threat could be posed to human health. However, there is a paucity of data on tick-borne zoonoses in Pakistan. In the current study, tick species infesting livestock across the Pakistan were catalogued, and molecular approaches were utilized to gain an insight into the microbiome associated with tick species. The results of our study revealed 19 tick species infesting livestock, and rich diversity of microbial communities associated with the ticks. Spotted Fever Group rickettsia, and *Theileria annulata* specific quantitative PCR assay confirmed the infection in Hyalomma tick species. To the best of our knowledge, this is the first detailed molecular study on tick species infesting livestock in Pakistan, and in depth vector competence studies should be conducted.

## Introduction

Pakistan, a predominantly farming nation, has an agriculture sector representing 20.9% of the country’s total gross domestic product and employs 43.4% of the country’s total workforce. According to the 2013/14 Pakistan Livestock Census [1], the livestock sector within the agricultural economy doubled from 25.3% in 1996 to 55%. The gross value of the livestock increased from $7.22 billion (Rs. 756.3 billion) in 2012/ 13 to $7.41 billion (Rs. 776.5 billion) in 2013/14, an increase of 2.7% as compared to the previous year. Gross production of milk in Pakistan increased from 47,895 million tons in 2011/12 to 50,990 million tons in 2013–14. Among the 8.4 million dairy-producing households, 51% own a herd of one to four animals, and 28% maintain five to ten animals [1]. Buffaloes and cows are the major milk-producing animals and 80% of the milk in Pakistan is produced by rural smallholders and commercial producers. The role of the livestock sector in the rural economy is crucial, as 30–35 million people in the rural population rely on this sector for their livelihoods.

Ticks and tick-borne diseases cause an estimated US $ 13.9 to 18.7 billion loss and an annual shortfall of approximately 3 billion pieces of hide and skin in cattle alone [2,3]. Ticks are known for their negative impact on livestock and human health through infestation and are capable of transmitting a wide range of pathogens including protozoans, viruses, and bacteria such as the spirochetes and rickettsiae. *Rhipicephalus*, *Haemaphysalis* (hereafter referred to as *Ha*. in species names), *Hyalomma* (hereafter referred to as *Hy*. in species names) and *Ornithodoros*, which are widely distributed throughout Pakistan, are the main tick genera infesting humans and animals [4,5]. A study in 1960 reported the presence of *Haemaphysalis cornupunctata* and *Ha*. *kashmirensis* in Pakistan [4]. *Hyalomma* and *Rhipicephalu*s tick species pose major threats to livestock production in Pakistan. The cattle tick *Rhipicephalu*s *microplus* is a competent vector of *Babesia bovis*, *B*. *bigemina*, and *Anaplasma marginale*, which cause tick fever in Pakistan and the rest of the world [6]. *Hyalomma* species are known vectors of *Theileria annulata*, a malaria like disease of animals [7]. Despite the pressing need for more information on the epidemiology of tick-borne zoonosis in Pakistan, there is a paucity of such data.

It has been reported that tick species simultaneously harbor a variety of pathogenic species and endosymbionts, and the communities of such organisms are known as pathobiomes and microbiomes, respectively [8–10]. The pathobiome is defined as pathogenic bacteria, virus or fungi within the community of the bacteria or biotic environment which itself can be described as subset of overall bacterial community (microbiome) which possesses or gain pathogenicity during the interaction within bacterial community. Previous microbial community descriptions have relied heavily on *in vitro* culture-based identification tools; however, the metagenomic approach offers a convenient alternative for obtaining microbial profiles. Specifically, pyrosequencing of partially amplified *16S rRNA* sequences has been used for studying the bacterial composition and diversity associated with many diverse biological organisms including *Ixodes ricinus*, *R*. *microplus*, *Amblyomma americanum*, *A*. *maculatum*, and *A*. *tuberculatum*, and neotropical tick species [11–15]. In fact, even though humans are considered “accidental hosts” of ticks, the rickettsial diseases transmitted by various arthropod vectors affect an estimated one billion people worldwide [16,17]. In Pakistan, an early study using serological assays reported the presence of rickettsial agents in ticks [5,18]. However, antigen conservation among the various rickettsial species makes it difficult to accurately identify rickettsial species using antibodies [19].

Limited information is available on the diversity of tick species that infest ruminants, their associated microbial diversity, and tick-borne pathogens in Pakistan. Therefore, the aim of this study was to survey the range of tick species and bacterial diversity in these ticks to facilitate better understanding of these species in Pakistan. To the best of our knowledge, this is the first detailed molecular study on tick species infesting livestock in Pakistan. We also investigated the presence of pathogenic rickettsial infections and the presence of the protozoan *T*. *annulata* in the tick species we collected.

## Methods

### Ethics statement

This study was carried out in accordance with the Manual for the Use of Animals of the Pakistan Veterinary Association. This protocol was approved by the Institutional Animal Care and Use Committees at each respective Pakistan-based institution (The University of Agriculture, Faisalabad, Sindh Agriculture University, Tando Jam, and Lasbela University of Agriculture, Lasbela).

### Study area, tick collection and processing

**Fig 1:**
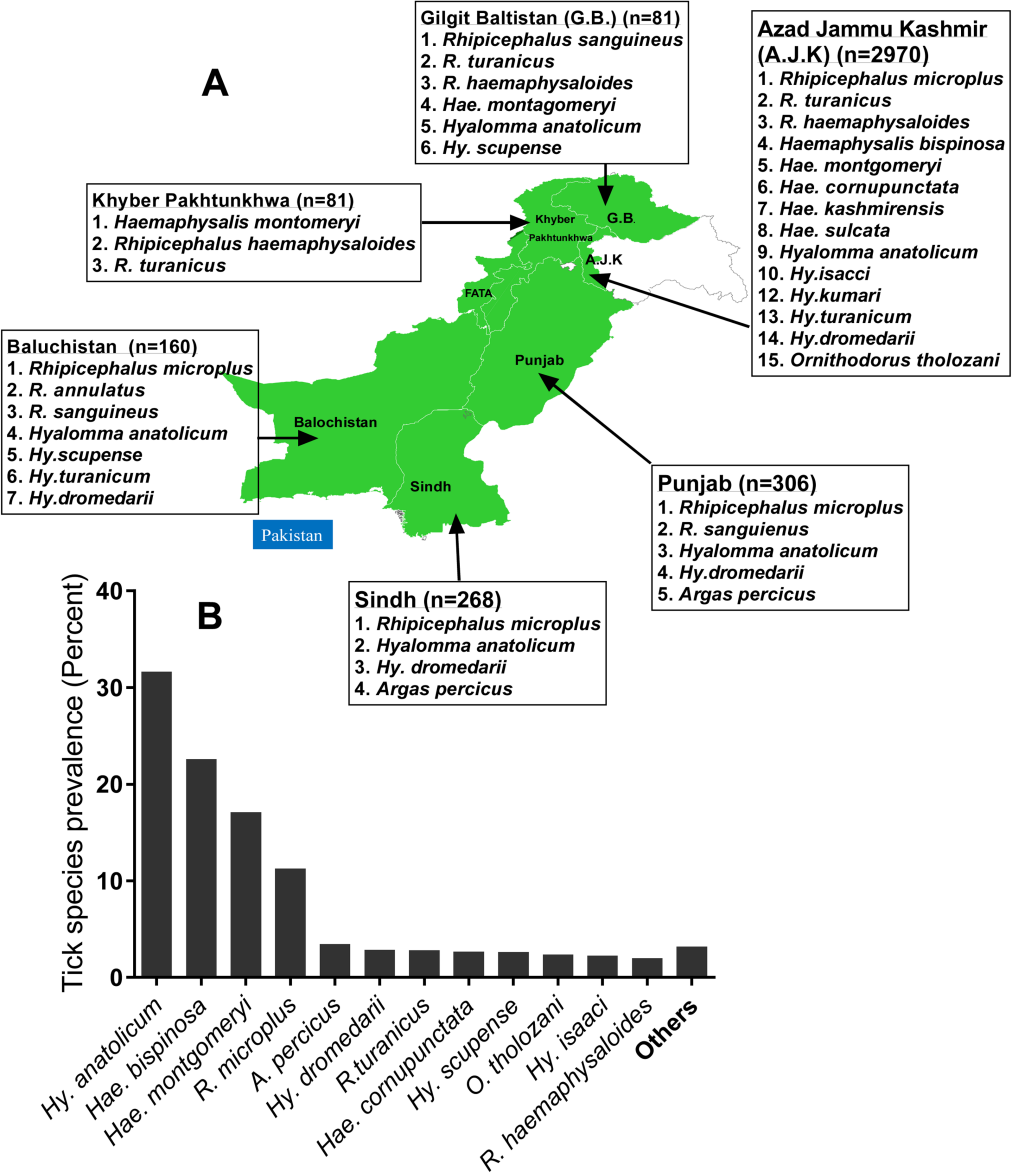
Tick species diversity infesting livestock of Pakistan. (A) Tick species prevalent in different geographic regions of Pakistan (B) Tick species prevalence in Pakistan. Species with less than 1% abundance are grouped as “others”; these include *Haemaphysalis kashmirensis* (0.98), *Hyalomma turanicum* (0.67), *Rhipicephalus sanguineus* (0.39), *Hae*. *sulcata* (0.34), *Hy*. *kumari* (0.10), *Hy*. *hussaini* (0.08), and *R*. *annulatus* (0.08). *Haemaphysalis* is abbreviated to *Hae*. *Hyalomma* is abbreviated to *Hy*. Ticks (~ 4000) were collected from different livestock and domestic animal hosts (cattle, buffalo, sheep, goat, camel, dog, cat and poultry) across the country.

A total of 3,866 ticks belonging to 19 species were collected from a variety of ruminant species from different geographic regions of Pakistan (S1 Table). The livestock pocket area of different provinces of Pakistan (Fig 1A) were visited in 2011–12 and tick infestation in livestock farm (Cattle, Buffalo, Sheep, Goat, Camel, Poultry) or domestic animals (Cat and dog) were assessed by veterinarian from University of Agriculture, Faisalabad; Sindh Agriculture University, and Lasbela University of Agriculture, Lasbela. Ticks were collected based on the livestock or domestic animal host (S1 Table) to understand tick species specific to host and further survey pathogenic bacteria or Theileria in ticks. This study was solely focused on the ticks, and tick-associated pathogens. Ticks attached to the animals were carefully removed using fine tweezers and then surface sterilized by rinsing them in distilled water followed by 100% ethanol to remove any surface bacteria and/or any host tissue. Ticks were stored in 70% ethanol and shipped from Pakistan to the University of Southern Mississippi for further analysis using the U.S. Department of Agriculture's Animal and Plant Health Inspection Service (permit # 11122050).

### Morphological identification of tick species

Tick identification was performed by an expert taxonomist (Dmitry A. Apanaskevich) at the United States National Tick Collection (USNTC) according to the criteria used in previously published reports [4,20–22]. All stages were examined on an Olympus SZX16 stereoscopic microscope and reference specimens from this study have been deposited in the USNTC at Georgia Southern University, USA.

### Genomic DNA extraction and pooling

Individual tick samples were cut into small pieces using a sterile scalpel and then homogenized in 200 μL of phosphate-buffered saline (pH 7.4) with a sterile micro-pestle. The individual tick homogenates were further disrupted by passage through a 27-guage needle attached to a 1 mL sterile syringe [23]. Genomic DNA was extracted from each individual whole tick homogenate using a DNeasy blood and tissue kit (Qiagen, Valencia, CA, USA) following the manufacturer’s protocol. The concentrations of the extracted genomic DNA samples were quantified using a Nanodrop ND-100 instrument. The extracted genomic DNA samples were stored at −20°C until further use. Prior to sequencing, the samples were pooled to survey the sequence diversity of the microbial communities because of the technical and financial burden of conducting a more complete analysis, despite the loss of statistical data. Fifteen sample pools were constructed from a variable number of ticks originating from ten different species and four different hosts (S2 Table). A total of 514 individual ticks of different species were screened for PCR identification of the spotted fever Rickettsia group (SFGR). Additionally, 387 *Hyalomma* ticks were screened for the presence of *Theileria* species infections.

### Bacterial profiling of ticks

Pyrosequencing analysis of tick DNA and analysis of the downstream sequencing data was performed as previously described [13]. Briefly, tick DNA samples were used for bacterial tag-encoded titanium amplicon pyrosequencing (bTETAP) [24]. The output used for analysis had an average read length of approximately 450-bp with the sequencing extending from the 27F 5′- GAG TTT GAT CNT GGC TCA G-3′ to 519R 5′-GTN TTA CNG CGG CKG CTG-3′ primers in relation to *Escherichia coli* 16S, extending across V1 and into the V3 ribosomal region (Research and Testing Laboratory, Lubbock, TX). A single-step 30-cycle PCR with HotStarTaq plus master mix kit (Qiagen) was used under the following conditions: 94°C for 3 min, followed by 32 cycles of 94°C for 30 s, 60°C for 40 s, and 72°C for 1 min, and a final elongation step at 72°C for 5 min. Following PCR, all amplicon products from the different samples were mixed to an equal concentration and purified using Agecourt Ampure beads (Agencourt Bioscience Corporation, MA, USA). Samples were sequenced utilizing Roche 454 FLX titanium instruments and reagents and following the manufacturer’s guidelines. The sequences were curated to obtain Q25 sequence data, which was processed using a proprietary analysis pipeline (www.mrdnalab.com) and the QIIME pipeline (www.qiime.org). All the sequences were trimmed to remove barcodes, primers, and short sequences under 200-bp in length. Sequences with ambiguous base calls and homopolymer runs exceeding 6-bp in length were deleted [25–27]. The taxonomic levels for the operational taxonomic unit (OTU) classifications were performed using the Basic Local Alignment Search Tool (BLASTn) program at the National Center for Biotechnology Information (NCBI, https://www.ncbi.nlm.nih.gov/) against the curated GreenGenes database [28] in QIIME 1.9 (http://qiime.org/) [29]. The taxonomic levels of the bacterial classes, family and genera were profiled across the tick species. All the raw sequences obtained were submitted to GenBank under the Pakistani Tick Microbiome Bioproject (PRJNA279069).

### Spotted fever group of *Rickettsia* (SFGR) detection

SFGR infections were detected using rickettsial outer membrane protein A (*rompA*) gene-specific primers in a nested PCR assay [23]. Briefly, RR190-70 (5′-ATGGCGAATATTTCTCCAAAA-3′) and RR190-701 (5′-GTTCCGTTAATGGCAGCATCT-3′) primers were used for primary PCR, while 190-FN1 (5′-AAGCAATACAACAAGGTC-3′) and 190-RN1 (5′-TGACAGTTATTATACCTC-3′) primers were used for nested PCR. In the primary reaction, 2.5 μL of DNA template (∼62.5 ng) was added to 12.5 μL of 2× PCR Master Mix (Promega, Madison, WI), 8 μL of nuclease-free water, and 1 μL of each primer (10μM). In the nested reaction, 12.5 μL of 2× PCR Master Mix, 8 μL of nuclease-free water, 1 μL of each nested primer (10μM), and 2.5 μL of the primary PCR reaction were used. PCRs were performed in a MyCycler Thermal Cycler (Bio-Rad Laboratories, USA) as follows: 1 cycle at 95°C for 3 min, 35 cycles of 95°C for 20 s, 46°C for 30 s, and 63°C for 60 s, and 1 cycle at 72°C for 7 min. The amplicons were separated on a 2% agarose gel containing ethidium bromide and then observed using a UV transilluminator. After electrophoresis, PCR products of 540-bp in length were excised from the agarose gel, and the DNA was extracted using a QIAquick DNA gel extraction kit (Qiagen). The purified DNA samples were sent to Eurofins MWG Operon (Huntsville, AL) for sequencing. The partial sequences obtained were checked against the NCBI BLAST program for rickettsial identification and the unique sequences were deposited in GenBank under accession numbers JX441089–JX441113 and KC245100–KC245101.

### *“Candidatus* Rickettsia amblyommii” quantification

*Candidatus* R. amblyommii was identified and quantified by targeting the gene encoding the rickettsial outer membrane protein B (*rompB*) in a quantitative PCR (qPCR) assay [30]. Briefly, *Candidatus* R. amblyommii genomic DNA (GenBank accession FJ455415, a gift from the Viral and Rickettsial Diseases Department at the Naval Medical Research Center, Silver spring, MD) was used to amplify *rompB* using the *rompB* gene-specific primers Ra477F (5'-GGTGCTGCGGCTTCTACATTAG-3'), Ra618R (5'-CTGAAACTTGAATAAATCCATTAGTAACAT-3'), and the *Candidatus* R. amblyommii specific-probe Ra532 (FAM-CGCGATCTCCTCTTACACTTGGACAGAATGCTTATCGCG-BHQ-1). The reaction mixture contained 0.5 μM of each primer, 0.4 μM of the probe, and 3 mM magnesium chloride in 12.5 μl of 2× TaqMan PCR master mix (Promega). The reaction mix was subjected to thermal cycling (CFX96 Real-time Detection System, BioRad Laboratories, CA) at 95°C for 2 min followed by 45 two-step cycles of 94°C for 5 s and 60°C for 30 s. The *Candidatus* R. amblyommii copy number was estimated using the standard curve generated from predetermined *rompB* DNA concentrations.

### PCR detection of *Piroplasma* species

*Piroplasma* spp. were PCR-detected using *Theileria* genus-specific primers that amplify the 18S ribosomal rRNA gene (Forward: 5′-GGT AAT TCC AGC TCC AAT AG-3′ and Reverse 5′-ACC AAC AAA ATA GAA CCA AAG TC-3′). The PCR mixture contained 25–35 ng of genomic DNA from the ticks, 400 nM of each primer, and PCR master mix (Biolab Inc.). The reaction mix was subjected to thermal cycling at 94°C for 3 min followed by 39 cycles of 94°C for 20 s, 48°C for 60 s, and 68°C for 30 s, and a final extension step at 68°C for 2 min. The amplicons obtained were isolated and purified using a gel purification kit (Qiagen), and the purified products were sequenced by Eurofins. The partial sequences obtained were subjected to the NCBI BLAST program for species identification of the piroplasma sequences.

### Quantification of *T*. *annulata*

*T*. *annulata* was quantified using a method described previously [31]. Briefly, *T*. *annulata* 18S rRNA gene-specific primers (Tann18SF: 5′-AGACCTTAACCTGCTAAATAGG-3′ and Tann18SR: 5′-CATCACAGACCTGTTATTGC-3′, 200 nM each) and 150 nM of the specific probe (FAM 5′-AAG[+T]TT[+C]TA[+C]TG[+T]CCCGTT-3′ BHQ1) were used in a 25 μl PCR mixture containing 2× One Taq PCR master mix (BioLabs, USA). The mixture was subjected to qPCR on a CFX96 instrument (BioRad Inc.) using cycling conditions of 50°C for 2 min, 95°C for 10 min and 40 cycles of 95°C for 15 s and 60°C for 1 min. Samples were analyzed in triplicate along with the three non-template controls on each plate. *T*. *annulata* quantification was performed using the standard curve derived from the cycle threshold values obtained from known 18S rRNA PCR concentrations.

### Data management

All the ticks were collected from livestock animals across the Pakistan and collected ticks were stored in 70% ethanol by veterinarian and students from University of Agriculture, Faisalabad; Sindh Agriculture University; and Lasbela University of Agriculture, Lasbela and shipped to University of Southern Mississippi. The tick vials were labelled with host species and geographical region of collection including the date and name of collector. Each tick was identified by taxonomist (Dmitry A. Apanaskevich) at the United States National tick collection (USNTC) and separated based on identified tick species from each original vial. Part of the identified specimen were deposited in the collection housed at USNTC. All the identified ticks were used for subsequent microbial and pathogenic bacterial identification and quantifications. All the data were generated at the University of Southern Mississippi and all the sequences generated by 16S rRNA and spotted fever group rickettsia detection were deposited in respective public repositories.

## Results

### Tick species identification

During the ecological survey of the ruminants in Pakistan, a total of 3,866 ticks belonging to 19 species were collected (S1 Table). These ticks included males (n = 1,330), females (n = 2,066), larvae (n = 570), and nymphs (n = 413) (S1 Table). Two soft tick species (*Argas persicus* and *Ornithodoros tholozani*) and 17 hard tick species (*Hy*. *bispinosa*, *Ha*. *cornupunctata*, *Ha*. *montgomeryi*, *Ha*. *sulcata*, *Ha*. *kashmerensis*, *Hy*. *anatolicum*, *Hy*. *dromedarii*, *Hy*. *isaaci*, *Hy*. *kumara*, *Hy*. *scupense*, *Hy*. *turanicum*, *Hy*. *hussaini*, *R*. *microplus*, *R*. *haemaphysaloides*, *R*. *sanguineus*, *R*. *turanicus*, *and R*. *annulatus*) were found (Fig 1). However, the following four tick species comprised over 80% of the total samples: *Hy*. *anatolicum* (n = 1,203), *Ha*. *bispinosa* (n = 853), *Ha*. *montgomeryi* (n = 641), and *R*. *microplus* (n = 416) (Fig 1).

Map of Pakistan is prepared from Information management unit, Food and Agriculture Organization of the United Nations, Pakistan.

### Microbial diversity in ticks

After curation, we obtained 58,194 sequences from 15 samples (average 3,879 sequences per sample) and these formed 544 unique OTUs. Profiling of the bacteria sampled from the various livestock species identified, in decreasing order of abundance, six main classes: Bacilli, ɤ-Proteobacteria, β-proteobacteria, Clostridia, α-proteobacteria and Actinobacteria (S3 Table). There were 30 bacterial families representing over 2% of the sequence reads obtained from the tick species. Overall, Oxalobacteraceae, Staphylococcaceae, Clostridiaceae, Enterobacteriaceae, Coxiellaceae, Rickettsiaceae, Streptococcaceae, and Lactobacillaceae were the predominant families (S1 Fig, S4 Table).

In the *R*. *microplus* ticks collected from cattle (group 1), Enterobacteriaceae was the most prevalent bacterial family. However, Rickettsiaceae, Oxalobacteraceae, and Micrococcaceae were abundant in the *R*. *turanicus* ticks infesting goats (group 2) (S1 Fig, S4 Table). In group 3, *Ha*. *cornupunctata* from sheep, and in group 4 *Ha*. *cornupunctata* from goats, contained Oxalobacteraceae, Enterobacteriaceae, Staphylococcaceae, but no Rickettsiaceae. *Ha*. *kashmerensis* from goats (group 5), *Ha*. *montgomeryi* from goats (group 6) and *Ha*. *montgomeryi* from buffaloes (group 7) were the dominant tick species for Rickettsiaceae along with Staphylococcaceae and Clostridiaceae, respectively (S1 Fig, S4 Table). In the *Ha*. *montgomeryi* ticks infesting cattle (group 8), Enterobacteriaceae, Oxalobacteraceae, and Staphylococcaceae were the dominant families, whereas Staphylococcaceae and Streptococcaceae were dominant in *Ha*. *bispinosa* from goats (group 9) (S1 Fig, S4 Table). Clostridiaceae solely dominated *Ha*. *bispinosa* removed from buffaloes (group 10), but *Hy*. *anatolicum* removed from cattle (group 11) and buffaloes (group 12) was dominated by Staphylococcaceae, Oxalobacteraceae, Burkholderiaceae, and Pseudomonades. Coxiellaceae solely dominated *Hy*. *scupense* infesting goats (group 13). Similarly, Lactobacillaceae and Staphylococcaceae bacterial families were dominant in *Hy*. *isaaci* blood-fed on cattle, whereas Oxalobacteraceae was found solely in the soft tick, *O*. *tholozani* from buffaloes (S1 Fig, S4 Table).

**Fig 2:**
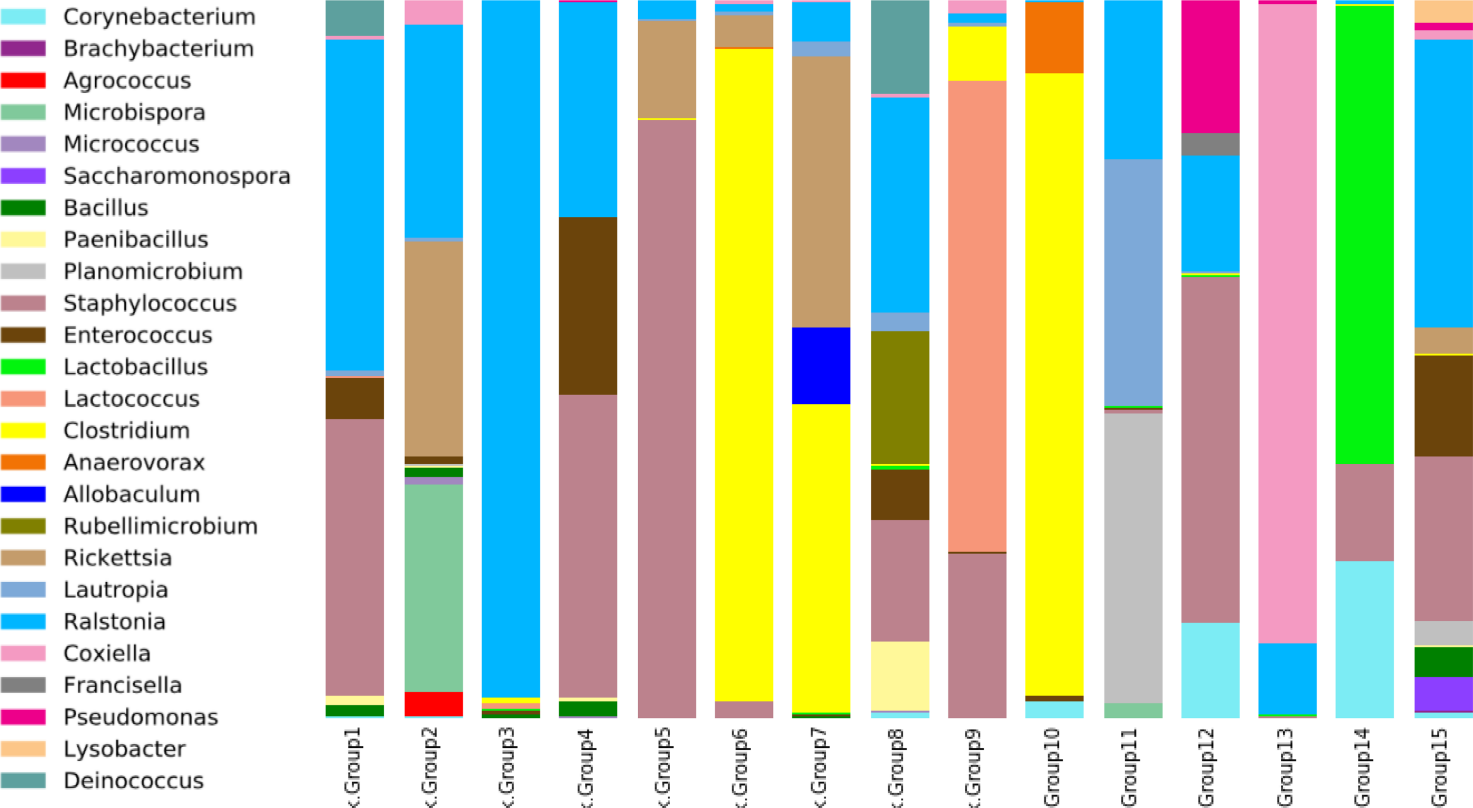
Bacterial diversity at the genus level in ticks from livestock in Pakistan. Group 1, *Rhipicephalus microplus* from cows; Group 2, *R*. *turanicus* from goats; Group 3, *Haemaphysalis cornupunctata* from sheep; Group 4, *Ha*. *cornupunctata* from goats; Group 5, *Ha*. *kashmerensis* from goats; Group 6, *Ha*. *montgomeryi* from goats; Group 7, *Ha*. *montgomeryi* from buffaloes; Group 8, *Ha*. *montgomeryi* from cows; Group 9, *Ha*. *bispinosa* from goats; Group 10, *Ha*. *bispinosa* from buffaloes; Group 11, *Hyalomma anatolicum* from cows; Group 12, *Hy*. *anatolicum* from buffaloes; Group 13, *Hy*. *scupense* from goats; Group 14, *Hy*. *isaaci* from cows; Group 15, *Ornithodoros tholozani* from buffaloes. Less than 2% of genera were removed during graph preparation. *Haemaphysalis* is abbreviated to *Ha*. *Hyalomma* is abbreviated to *Hy*.

The dominant bacterial genus was *Ralstonia*. It was present in all the tick species, comprising up to 97% of the total number of sequences for *Ha*. *cornupunctata* collected from sheep (group 3), but as low as 0.3% in *Ha*. *bispinosa* collected from buffaloes (group 10) (Fig 2). The *Clostridium* genus was most prevalent (>80%) in *Ha*. *montgomeryi* from goats (group 6), buffaloes (group 7), and in *Ha*. *bispinosa* from goats (group 9) and buffaloes (group 10) (Fig 2). *Corynebacterium* was dominant in *Ha*. *bispinosa* and *Hy*. *anatolicum* from buffaloes (group 12). *Staphylococcus* was most abundant in *R*. *microplus* from cattle (group 1), in *Ha*. *cornupunctata* (group 4) and *Ha*. *kashmerensis* from goats (group 5) and in *Hy*. *anatolicum* from buffaloes (group 12) (Fig 2). The *Rickettsia* genus was dominant (3–40%) in *R*. *turanicus* (group 2) and *Ha*. *cornupunctata* from goats. Similarly, *Rickettsia* was dominant in *Ha*. *montgomeryi* from goats (group 6) and buffaloes (group 7), and in *Hy*. *anatolicum* from buffaloes (group 12). Interestingly, we did not observe *Rickettsia* in any other tick species (Fig 2, S5 Table). *Coxiella* was the dominant genus in *Hy*. *scupense* collected from goats (group 13, while *Francisella* was present in *Hy*. *anatolicum* (group 12) (Fig 2).

### SFGR and *Rickettsia amblyommii*

A total of 514 ticks were individually screened and 54 (54/514, 10%) rickettsial fragments were identified (Table 1) based on the partial rickettsial *ompA* sequences. Twenty-one tick samples were identified as “*Candidatus* Rickettsia amblyommii” which is an infection rate of 4% (21/514) of the total number of ticks tested (Table 1). Among the tested ticks, DNA isolated from *Hy*. *isaaci*, *R*. *turanicus* and *R*. *sanguineus* was not PCR-amplifiable for SFGR (Table 1). The *Candidatus* R. amblyommii-infected tick DNAs were further verified by qPCR by specific amplification of the rickettsial *rompB* gene and the copy numbers ranged from 40–10,497 (Table 2). The copy numbers for *Candidatus* R. amblyommii in the *Hy*. *anatolicum* ticks removed from a variety of ruminants varied from as low as 40, to a maximum of over 10,000 (Table 2). Similarly, *Hy*. *dromoderii* was infected with *Candidatus* R. amblyommii via blood feeding on goats and camels (Table 2).

**Table 1:** Ticks tested for SFG Rickettsia.

Ticks	Number of ticks tested	SFG Rickettsia(*ompA*)
*Hyalomma anatolicum*	377	21
*Hyalomma dromedarii*	49	7
*Hyalomma kumari*	2	2
*Hyalomma scupense*	6	1
*Hyalomma isaacii*	2	0
*Haemaphysalis montgomeryi*	31	8
*Haemaphysalis bispinosa*	28	4
*Haemaphysalis cornupunctata*	6	1
*Haemaphysalis kashmerensis*	6	2
*Rhipicephalus microplus*	43	9
*Rhipicephalus turanicus*	8	0
*Rhipicephalus haemphasyalis*	4	1
*Rhipicephalus sanguineus*	12	0
Total	574	54

**Table 2:** Detection of spotted fever group of Rickettsia and quantification of *R*. *amblyommii* in ticks from livestock.

Host	Identified tick species	SFGR (*ompA*)	GenBank *ompA* Acc #	% Nucleotide identity	*R*. *amblyommii* copies/μL (*ompB* qPCR)
Buffalo	*Hyalomma anatolicum*	*Candidatus R*. *amblyommii*	JX441091 JX441092 JX441095 JX441115	100	40–10497
Cattle	*Hyalomma anatolicum*	*Candidatus R*. *amblyommii*	JX441098 JX441099	100	11406- 11095
		*RE H*. *anatolicum*	JX441100		-
	*Rhipicephalus microplus*	*Candidatus R*. *amblyommii*	JX441089 JX441093	100	45–74
		*RE R*. *microplus*	JX441090 JX441094	^Δ^NA	-
Sheep	*Hyalomma anatolicum*	*Candidatus R*. *amblyommii*	JX441105 JX441106 JX441107 JX441108	99–100	54–4766
	*Rhipicephalus* *microplus*	RE *R*. *microplus*	JX441096 JX441097	^Δ^NA	-
Goat	*Hyalomma anatolicum*	*Candidatus R*. *amblyommii*	JX441109 JX441110	100	2609–10497
	*Hyalomma* *dromoderii*	*RE H*. *dromedarii*	KC245100	^Δ^NA	-
		*Candidatus R*. *amblyommii*	JX441114 JX441111 JX441112	99–100	43–7373
	*Rhipicephalus* *microplus*	*RE R*. *microplus*	JX441116	^Δ^NA	-
Donkey	*Hyalomma anatolicum*	*Candidatus R*. *amblyommii*	JX441101 JX441102	99–100	3793–5668
Camel	*Hyalomma anatolicum*	*Candidatus R*. *amblyommii*	JX441103	99	5108
	*Hyalomma* *dromoderii*	*Candidatus R*. *amblyommii*	JX441113	100	61
		*RE H*. *dromedarii*	JX441104 KC245101	^Δ^NA	-

^Δ^NA indicates–identified in this study

### Detection and quantification of *T*. *annulata*

In total, 387 randomly selected *Hyalomma* ticks were individually tested for infection with the pathogenic protozoan, *Theileria*, using *Theileria* 18S rRNA gene-specific primers. Table 3 shows the results for PCR amplification of DNA from 22 *Hy*. *anatolicum* and *Hy*. *dromedarii* ticks. DNA sequencing of the *Theileria*-specific 18S rRNA PCR amplicons revealed *Theileria-* or *Babesia*-like sequences based on the closest homology (Table 3). The *T*. *annulata*-specific qPCR assay using a specific probe was used to genetically identify *T*. *annulata* in the screened tick samples and, surprisingly, 19 out of 22 were positive with an infection rate varying from 100–3887 copies/μL (Table 3).

**Table 3:** Piroplasm detection and quantification of *Theileria annulata* in *Hyalomma* ticks from livestock in Pakistan.

Ticks	Closest Homology (n = 387) (PCR amplicon sequences)	qPCR of *T*. *annulata* (Mean Copy no./μL)
*Hy*. *anatolicum*	*Babesia bigemina*	233
	*Theileria luwenshuni*	1125
	*Theileria sp*.	3022
	*Theileria sp*.	281
	*Theileria annulata*	540
	*Theileria annulata*	3887
	*Babesia bigemina*	396
	*Theileria sp*.	226
	*Babesia canis canis*	217
	*Babesia bigemina*	173
	*Babesia bigemina*	155
	*Babesia bigemina*	159
	*Babesia bigemina*	226
	*Theileria cf*. *velifera*	140
	*Theileria cf*. *velifera*	183
	*Babesia bigemina*	126
	*Babesia gibsoni*	196
*Hy*. *dromedarii*	*Theileria lestoquardi*	126
	*Babesia occultans*	173

### Co-infection in ticks

The co-occurrence/co-infection of tick pathogens, *Theileria* and *Babesia* were reported in this study in *Hyalomma anatolicum* and *Hyalomma dromedarii* ticks (Table 3). The primers which amplify both piroplasma species was selected to decipher presence of both in ticks using PCR methods. The piroplasma species were targeted in 17 *Hy*. *anatolicum* species, and nine of these ticks detected *Babesia* by PCR assay. A further testing of these ticks revealed *Theileria annulata* amplicons as tested by qPCR specific assay suggesting possible co-occurrence or co-infection of *Babesia* and *Theileria* species. Intriguingly, only two *Hy*. *dromerdarii* ticks tested for piroplasma infection, and one showed the co-infection both piroplasma species.

## Discussion

Tick infestations cause substantial blood losses from livestock and can also transmit severe diseases such as theileriosis and babesiosis [7]. In Pakistan, the impact of ticks and tick-borne infectious diseases in the livestock sector and public health requires urgent investigation. The diseases transmitted by ticks to livestock inflict devastating losses to the livestock sector in rural Pakistan. Tick infestations, and tick-borne pathogens significantly decrease the production of milk, meat, wool, and hide. In the present study, we have reported on the presence of 19 different tick species prevalent in different ecological and geographical regions of Pakistan (Fig 1, S1 Table). We found that tick infestation levels varied across the different localities we tested in Pakistan, a finding probably resulting from ecological variation in the regions. The highest diversity of tick species was found in the Azad Jammu and Kashmir region (15 species), while the lowest was in Khyber Pakhtunkhwa (3 species) (S1 Table). The high diversity of tick species in Baluchistan, Azad Jammu and Kashmir and Gilgit-Baltistan was possibly caused by the nomadic life style in these regions, while in Punjab, Sindh, and Khyber Pakhtunkhwa the animal husbandry is known to be well established. The poultry tick, *Argas persicus*, was only found in Punjab, because ticks were also collected from poultry in this region (S1 Table. Overall, *Hy*. *anatolicum*, *Ha*. *bispinosa*, *Ha*. *montgomeryi* and *R*. *microplus* were the dominant tick species infesting livestock in the different ecological regions of Pakistan that we investigated (S1 Table). Five species of *Rhipicephalus* and *Haemaphysalis* each, seven species of *Hyalomma* and one each of *Argas* and *Ornithodorus* were found to be actively blood feeding on livestock. Interestingly, *Amblyomma* and *Ixodes* infestation has been reported previously in Pakistan [6], but we did not find either of these ticks in our study. *R*. *microplus* and four other species of this genus are one-host ticks known to transmit *B*. *bovis*, *B*. *bigemina*, *A*. *marginale* and spirochetes [32]. *Hy*. *anatolicum* is a three-host tick known to vector *T*. *annulata* [33]; other species of the same genus with two-host life cycles also known to transmit *Theileria* spp. include *Hy*. *dromedarii*, *Hy*. *scupense*, *Hy*. *isaaci*, *Hy*. *kumari*, *Hy*. *hussaini*, and *Hy*. *turanicum*. The *Haemaphysalis* species described by Hoogstraal and Anastos in 1968 [34] were identified as *Ha*. *cornupunctata* and *Ha*. *kashmirensis* from that region [4], while *Ha*. *bispinosa*, *Ha*. *montagomeryi*, and *Ha*. *sulcata* were found in west Pakistan and the Himalayan region. Two soft tick species were identified as *O*. *tholozani*, a competent vector of the Borrelia spirochete [35], while the poultry tick *A*. *persicus*, which can cause paralysis in poultry, birds was reported in another study [36].

Among the ticks collected and identified from livestock across Pakistan, the individually extracted DNAs were pooled by the species and livestock host they were removed from (S2 Table). Increasing the knowledge base about the bacterial species present in different tick species will yield important information about the possible risks to livestock the bacteria may present. The differences in microbial diversity among ticks were not considered substantial based on the tick genus, or the host from which they were collected, or the host type (single, double or multiple host system). However, ticks can harbor potential pathogens such as *Candidatus* R. amblyommii (Table 2) and the animal pathogen, *T*. *annulata* (Table 3). Microbial diversity in ticks plays a significant role in pathogen transmission, vector competence [37–39], tick reproductive fitness [40], along with other unidentified roles. The most dominant bacterial genera in the tick species were *Ralstonia*, *Clostridium*, *Corynebacterium* and *Staphylococcus*; the ticks probably obtained them from livestock skin and fur and they are often identified in other tick microbiome studies [11,12,15]. The variability of the bacterial profile within the different tick genera probably results from differences in the host genotype, health status, or their ecological location (Fig 2, S2 Table). Current study precluded the tick-borne pathogen detection in the host animals, and solely focused on the prevalence of tick infestation, and pathogens associated with the tick-vectors from different geographical regions of Pakistan (Fig 1B). Presumably, the blood meal from the infected livestock species is the source of tick infection, and pathogens residing inside tick hosts can modulate microbiome of ticks, and this study did not take in account this aspect [41]. *Rickettsia*, *Francisella* and *Coxiella* were the most important tick bacterial species that were identified associated with tick species infesting livestock in Pakistan (S5 Table) [42]. Intriguingly, *Rickettsia* has been shown to be maintained in the tick population via transovarial transmission [43,44]. This study provides an insight into the baseline information of tick species prevalence and pathobiome diversity. This information provides important clues for future studies aimed at the prevention of neglected tick and tick-borne infectious diseases in the region.

The presence of *Rickettsia* was further explored by PCR amplification of the SFGR-specific *ompA* gene [45]. The sequence homology of the amplicons was closest to *Candidatus* R. amblyommii and was further verified using an *Candidatus* R. amblyommii-specific qPCR assay [15,30]. The pathogenicity of *Candidatus* R. amblyommii has not yet been determined, but it is known to modulate the pathogenicity of other pathogens [46]. The presence of this rickettsial agent in *Hyalomma* and *R*. *microplus* (Table 2) possibly influences their vector competence as they are known to transmit theileriosis, babesiosis, and anaplasmosis [7,47,48].

Further, the *Hyalomma* ticks we collected were screened for piroplasma species using a PCR targeting the Theileria *18S rRNA* gene. Analysis of the resulting amplicons identified sequences with close homology to those from *Theileria* or *Babesia* (Table 3). Because of this ambiguity, we used a more definitive qPCR assay with a probe-based real-time PCR to validate the presence and infection level of *T*. *annulata* within the infected ticks (Table 3). The infection status of the livestock host was not determined, but the *Theileria* infection of the Hyalomma ticks removed from the bovine hosts highlights the possibility of an exchange of infection to and from the livestock. Bovine tropical theileriosis is vectored by *Hyalomma* species (e.g. *Hy*. *anatolicum*, *Hy*. *dromedarii*, *Hy*. *kumari*, and *Hy*. *scupense*), and is considered an economically important disease in cattle, resulting in high morbidity and economic losses [7]. Overall, our results portray the picture of tick host as “the microbe zoo”. The tick vectors are co-infected with a variety of diverse pathogenic and non-pathogenic symbionts. Characterization of microbial interactions within the tick host in multiple infections pose a gigantic scientific challenge for understanding the epidemiology of tick-borne infectious diseases. Interestingly, co-infections of pathogenic microbes within ticks is a complex phenomenon because tick-borne pathogens interact with both mammalian hosts, and arthropod vectors. An elegant study conducted on the field collected Ixodes ricinus supported the concept of co-infection of pathogens and symbionts as a rule in the ticks [49]. Our results showed the co-infection of *Babesia* and *Theileria* in *Hyalomma* ticks. A 50% chance of ticks infected with both piroplasma species can be predicted. The co-infection of piroplasma species depends upon the susceptibility of host species during tick attachment, tick’s vectorial capacity, pathogens’ ability to replicate within tick vector in competitive environment in tick’s midgut, salivary glands, and many ecological parameters of pathogen emergence and host factors. The study of *Babesia microti* coinfection with *Borrelia burgdorferi* in *Ixodes* ticks favors transmission of *B*. *microti* in areas endemic to Lyme disease despite low ecological fitness [50]. The domestic cattle survey for *Babesia* or *Theileria* infection reported mixed infection for both piroplasma species infer the possibility of transmission chances for both pathogenic species in cattle from *Hyalomma* species in Pakistan [51]. This study opens a new avenue of research to characterize the pattern of interactions among different tick-borne pathogens within tick vectors and mammalian hosts. Further experimental co-infections should elucidate the underlying mechanisms shaping the community ecology of co-infections inside the tick vector.

### Conclusions

In this study, we surveyed ticks from farm and domestic livestock holder’s animals in different ecological locations across Pakistan. We identified 19 different tick species representing three important hard ticks, *Hyalomma*, *Rhipicephalus* and *Haemaphysalis*, and two soft tick species, *Argas* and *Ornithodorus*. Bacterial pathogens in the ticks were assessed using next generation sequencing, which successfully profiled the bacteria from the different tick species, and we focused on validating the Rickettsial agents present in the ticks using this technique. The Rickettsial agent, *Candidatus* R. amblyommii, was identified and quantified. *Hyalomma* ticks were screened for the presence of the causative agent of bovine theileriosis, *T*. *annulata*, the levels of which within the ticks were also quantified.

## Supporting information

S1 Fig Bacterial diversity by class (top) and family (bottom) in ticks from livestock in Pakistan. Group 1, *Rhipicephalus microplus* from cows; Group 2, *R*. *turanicus* from goats; Group 3, *Haemaphysalis cornupunctata* from sheep; Group 4, *Ha*. *cornupunctata* from goats; Group 5, *Ha*. *kashmerensis* from goats; Group 6, *Ha*. *montgomeryi* from goats; Group 7, *Ha*. *montgomeryi* from buffaloes; Group 8, *Ha*. *montgomeryi* from cows; Group 9, *Ha*. *bispinosa* from goats; Group 10, *Ha*. *bispinosa* from buffaloes; Group 11, *Hyalomma anatolicum* from cows; Group 12, *Hy*. *anatolicum* from buffaloes; Group 13, *Hy*. *scupense* from goats; Group 14, *Hy*. *isaaci* from cows; and Group 15, *Ornithodoros tholozani* from buffaloes. Less than 2% of the species were removed during graph preparation. *Haemaphysalis* is abbreviated to *Ha*. *Hyalomma* is abbreviated to *Hy*.
(DOCX)

S1 Table Tick species diversity in Pakistan. (XLSX)

S2 Table Sample collection and processing information. (XLSX)

S3 Table Taxonomy summary (genera level). (HTML)

S4 Table Taxonomy summary (class level). (HTML)

S5 Table Taxonomy summary (family level). (HTML)
