# Supplementary material for: A study of ticks and tick-borne livestock pathogens in Pakistan
Source: PLoS Negl Trop Dis. 2017 Jun 26;11(6):e0005681. doi: 10.1371/journal.pntd.0005681 (PMC5501686; doi:10.1371/journal.pntd.0005681)
Supplement: S1 Fig — Group 1, Rhipicephalus microplus from cows; Group 2, R. turanicus from goats; Group 3, Haemaphysalis cornupunctata from sheep; Group 4, Ha. cornupunctata from goats; Group 5, Ha. kashmerensis from goats; Group 6, Ha. montgomeryi from goats; Group 7, Ha. montgomeryi from buffaloes; Group 8, Ha. montgomeryi from cows; Group 9, Ha. bispinosa from goats; Group 10, Ha. bispinosa from buffaloes; Group 11, Hyalomma anatolicum from cows; Group 12, Hy. anatolicum from buffaloes; Group 13, Hy. scupense from goats; Group 14, Hy. isaaci from cows; and Group 15, Ornithodoros tholozani from buffaloes. Less than 2% of the species were removed during graph preparation. Haemaphysalis is abbreviated to Ha. Hyalomma is abbreviated to Hy. (DOCX) [file pntd.0005681.s001.docx]

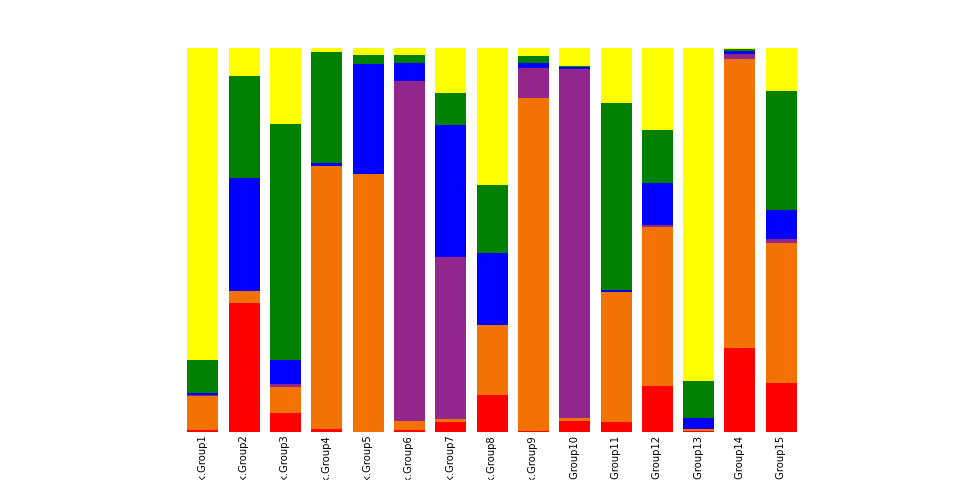

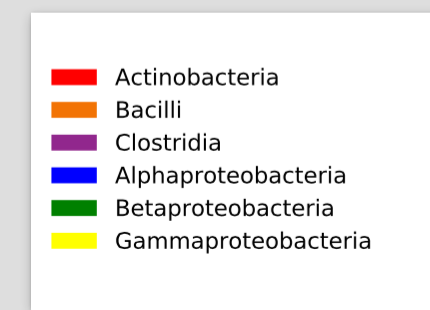

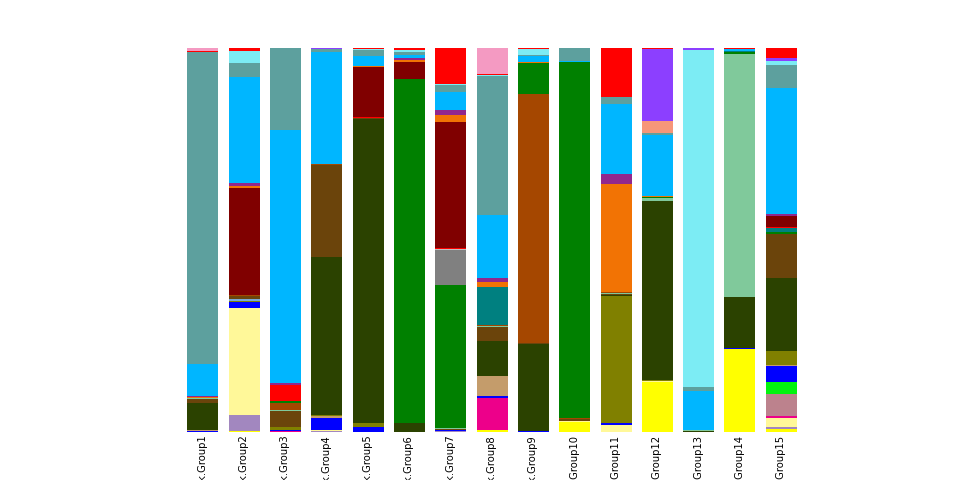

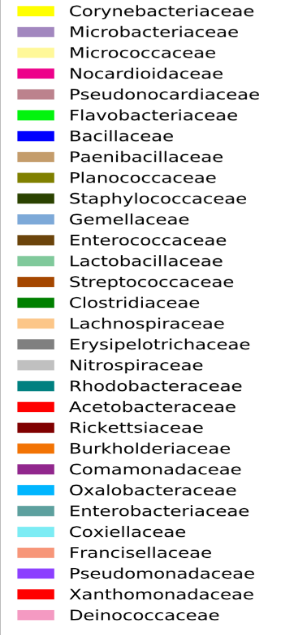


Figure S1. Bacterial diversity by class (top) and family (bottom) in ticks from livestock in Pakistan. Group 1, *Rhipicephalus microplus* from cows; Group 2, *R. turanicus* from goats; Group 3, *Haemaphysalis cornupunctata* from sheep; Group 4, *Ha. cornupunctata* from goats; Group 5, *Ha. kashmerensis* from goats; Group 6, *Ha. montgomeryi* from goats; Group 7, *Ha. montgomeryi* from buffaloes; Group 8*, Ha. montgomeryi* from cows; Group 9, *Ha. bispinosa* from goats; Group 10, *Ha. bispinosa* from buffaloes; Group 11, *Hyalomma anatolicum* from cows; Group 12, *Hy. anatolicum* from buffaloes; Group 13, *Hy. scupense* from goats; Group 14, *Hy. isaaci* from cows; and Group 15, *Ornithodoros tholozani* from buffaloes. Less than 2% of the species were removed during graph preparation. *Haemaphysalis* is abbreviated to *Ha.* *Hyalomma* is abbreviated to *Hy.*
