# Supplementary material for: A study of ticks and tick-borne livestock pathogens in Pakistan
Source: PLoS Negl Trop Dis. 2017 Jun 26;11(6):e0005681. doi: 10.1371/journal.pntd.0005681 (PMC5501686; doi:10.1371/journal.pntd.0005681)
Supplement: S3 Table — (HTML) [file pntd.0005681.s004.html]

Taxa Summaries


|  |  |
| --- | --- |
|  | |
| Taxonomy Summary. Current Level: | |
| View Figure (.pdf)  View Legend (.pdf) |  |
|  |


|  |
| --- |
| View Table (.txt) |

|  |  |  |  |  |  |  |  |  |  |  |  |  |  |  |  |  |  |
| --- | --- | --- | --- | --- | --- | --- | --- | --- | --- | --- | --- | --- | --- | --- | --- | --- | --- |
|  | | Total | Tick.Group1 | Tick.Group2 | Tick.Group3 | Tick.Group4 | Tick.Group5 | Tick.Group6 | Tick.Group7 | Tick.Group8 | Tick.Group9 | Tick.Group10 | Tick.Group11 | Tick.Group12 | Tick.Group13 | Tick.Group14 | Tick.Group15 |
| Legend | Taxonomy | % | % | % | % | % | % | % | % | % | % | % | % | % | % | % | % |
|  | Actinobacteria | 7.0% | 0.5% | 33.7% | 5.0% | 0.8% | 0.0% | 0.5% | 2.6% | 9.6% | 0.2% | 2.9% | 2.5% | 11.9% | 0.4% | 21.9% | 12.7% |
|  | Bacilli | 30.2% | 8.8% | 3.0% | 6.8% | 68.3% | 67.1% | 2.3% | 0.7% | 18.3% | 86.7% | 0.8% | 34.1% | 41.6% | 0.5% | 75.2% | 36.4% |
|  | Clostridia | 15.5% | 0.3% | 0.0% | 0.7% | 0.0% | 0.1% | 88.7% | 42.3% | 0.1% | 8.0% | 90.9% | 0.0% | 0.3% | 0.0% | 1.5% | 1.1% |
|  | Alphaproteobacteria | 9.6% | 0.4% | 29.4% | 6.3% | 0.9% | 28.6% | 4.8% | 34.3% | 18.6% | 1.3% | 0.5% | 0.5% | 11.0% | 2.7% | 0.6% | 7.8% |
|  | Betaproteobacteria | 17.5% | 8.8% | 26.7% | 61.7% | 29.0% | 2.3% | 2.0% | 8.1% | 17.9% | 1.8% | 0.3% | 48.6% | 13.9% | 9.8% | 0.5% | 30.9% |
|  | Gammaproteobacteria | 20.2% | 81.2% | 7.3% | 19.7% | 0.9% | 1.9% | 1.9% | 11.8% | 35.6% | 2.0% | 4.6% | 14.3% | 21.3% | 86.6% | 0.3% | 11.1% |
