# Supplementary material for: A study of ticks and tick-borne livestock pathogens in Pakistan
Source: PLoS Negl Trop Dis. 2017 Jun 26;11(6):e0005681. doi: 10.1371/journal.pntd.0005681 (PMC5501686; doi:10.1371/journal.pntd.0005681)
Supplement: S4 Table — (HTML) [file pntd.0005681.s005.html]

Taxa Summaries


|  |  |
| --- | --- |
|  | |
| Taxonomy Summary. Current Level: | |
| View Figure (.pdf)  View Legend (.pdf) |  |
|  |


|  |
| --- |
| View Table (.txt) |

|  |  |  |  |  |  |  |  |  |  |  |  |  |  |  |  |  |  |
| --- | --- | --- | --- | --- | --- | --- | --- | --- | --- | --- | --- | --- | --- | --- | --- | --- | --- |
|  | | Total | Tick.Group1 | Tick.Group2 | Tick.Group3 | Tick.Group4 | Tick.Group5 | Tick.Group6 | Tick.Group7 | Tick.Group8 | Tick.Group9 | Tick.Group10 | Tick.Group11 | Tick.Group12 | Tick.Group13 | Tick.Group14 | Tick.Group15 |
| Legend | Taxonomy | % | % | % | % | % | % | % | % | % | % | % | % | % | % | % | % |
|  | Corynebacteriaceae | 2.6% | 0.0% | 0.3% | 0.0% | 0.0% | 0.0% | 0.0% | 0.0% | 0.5% | 0.0% | 2.6% | 0.1% | 12.9% | 0.0% | 21.7% | 0.7% |
|  | Microbacteriaceae | 0.4% | 0.0% | 4.2% | 0.0% | 0.2% | 0.0% | 0.0% | 0.3% | 0.0% | 0.1% | 0.0% | 0.0% | 0.0% | 0.0% | 0.0% | 0.7% |
|  | Micrococcaceae | 2.2% | 0.0% | 27.8% | 0.0% | 0.3% | 0.0% | 0.0% | 0.0% | 0.0% | 0.0% | 0.2% | 1.8% | 0.5% | 0.0% | 0.0% | 2.3% |
|  | Nocardioidaceae | 0.6% | 0.0% | 0.0% | 0.1% | 0.1% | 0.0% | 0.0% | 0.0% | 8.5% | 0.0% | 0.0% | 0.0% | 0.0% | 0.0% | 0.0% | 0.4% |
|  | Pseudonocardiaceae | 0.4% | 0.0% | 0.0% | 0.0% | 0.0% | 0.0% | 0.0% | 0.0% | 0.0% | 0.0% | 0.0% | 0.0% | 0.0% | 0.0% | 0.0% | 5.8% |
|  | Flavobacteriaceae | 0.2% | 0.0% | 0.0% | 0.0% | 0.0% | 0.0% | 0.0% | 0.0% | 0.0% | 0.0% | 0.0% | 0.0% | 0.0% | 0.0% | 0.0% | 3.1% |
|  | Bacillaceae | 0.8% | 0.3% | 1.4% | 0.4% | 3.1% | 1.3% | 0.0% | 0.1% | 0.5% | 0.1% | 0.0% | 0.6% | 0.0% | 0.0% | 0.0% | 4.3% |
|  | Paenibacillaceae | 0.4% | 0.3% | 0.2% | 0.0% | 0.5% | 0.0% | 0.0% | 0.0% | 5.1% | 0.0% | 0.0% | 0.0% | 0.0% | 0.0% | 0.0% | 0.2% |
|  | Planococcaceae | 2.7% | 0.0% | 0.2% | 0.8% | 0.1% | 0.9% | 0.0% | 0.0% | 0.0% | 0.0% | 0.0% | 33.1% | 0.0% | 0.0% | 0.0% | 3.8% |
|  | Staphylococcaceae | 15.3% | 7.0% | 0.1% | 0.0% | 41.1% | 79.4% | 2.1% | 0.1% | 9.0% | 22.6% | 0.0% | 0.3% | 46.7% | 0.3% | 13.4% | 18.9% |
|  | Gemellaceae | 0.0% | 0.0% | 0.3% | 0.0% | 0.0% | 0.0% | 0.0% | 0.0% | 0.0% | 0.0% | 0.0% | 0.0% | 0.0% | 0.0% | 0.0% | 0.0% |
|  | Enterococcaceae | 3.2% | 1.0% | 1.0% | 4.1% | 24.2% | 0.1% | 0.0% | 0.2% | 3.7% | 0.2% | 0.6% | 0.2% | 0.1% | 0.0% | 0.0% | 11.4% |
|  | Lactobacillaceae | 4.4% | 0.3% | 0.0% | 0.1% | 0.0% | 0.0% | 0.0% | 0.3% | 0.1% | 0.0% | 0.0% | 0.3% | 0.7% | 0.3% | 63.3% | 0.1% |
|  | Streptococcaceae | 4.8% | 0.0% | 0.1% | 1.8% | 0.1% | 0.0% | 0.0% | 0.0% | 0.3% | 64.9% | 0.3% | 0.1% | 0.0% | 0.0% | 0.0% | 0.0% |
|  | Clostridiaceae | 15.3% | 0.0% | 0.0% | 0.7% | 0.0% | 0.1% | 89.6% | 37.3% | 0.1% | 8.1% | 92.7% | 0.0% | 0.4% | 0.0% | 0.3% | 0.5% |
|  | Lachnospiraceae | 0.0% | 0.0% | 0.0% | 0.0% | 0.0% | 0.0% | 0.1% | 0.0% | 0.0% | 0.0% | 0.0% | 0.0% | 0.0% | 0.0% | 0.0% | 0.0% |
|  | Erysipelotrichaceae | 0.6% | 0.0% | 0.0% | 0.0% | 0.0% | 0.0% | 0.0% | 9.2% | 0.0% | 0.0% | 0.0% | 0.0% | 0.0% | 0.0% | 0.0% | 0.0% |
|  | Nitrospiraceae | 0.0% | 0.0% | 0.1% | 0.0% | 0.0% | 0.0% | 0.0% | 0.1% | 0.1% | 0.0% | 0.0% | 0.0% | 0.0% | 0.1% | 0.0% | 0.0% |
|  | Rhodobacteraceae | 0.7% | 0.0% | 0.0% | 0.0% | 0.0% | 0.0% | 0.0% | 0.0% | 9.9% | 0.0% | 0.0% | 0.0% | 0.0% | 0.0% | 0.2% | 0.9% |
|  | Acetobacteraceae | 0.3% | 0.0% | 0.0% | 4.1% | 0.0% | 0.3% | 0.0% | 0.4% | 0.0% | 0.0% | 0.0% | 0.0% | 0.0% | 0.0% | 0.0% | 0.2% |
|  | Rickettsiaceae | 5.2% | 0.0% | 27.8% | 0.0% | 0.0% | 12.9% | 4.4% | 32.6% | 0.0% | 0.0% | 0.0% | 0.0% | 0.0% | 0.0% | 0.0% | 2.9% |
|  | Burkholderiaceae | 2.4% | 0.1% | 0.6% | 0.0% | 0.0% | 0.3% | 0.6% | 2.1% | 1.3% | 0.3% | 0.0% | 28.2% | 0.2% | 0.0% | 0.1% | 0.1% |
|  | Comamonadaceae | 0.5% | 0.1% | 0.7% | 0.4% | 0.0% | 0.1% | 0.4% | 1.3% | 1.1% | 0.0% | 0.0% | 2.7% | 0.0% | 0.0% | 0.0% | 0.5% |
|  | Oxalobacteraceae | 15.6% | 8.4% | 27.7% | 66.1% | 29.3% | 2.5% | 1.0% | 4.6% | 16.2% | 1.5% | 0.3% | 18.3% | 15.7% | 10.0% | 0.4% | 32.9% |
|  | Enterobacteriaceae | 10.9% | 81.3% | 3.6% | 21.2% | 0.7% | 1.6% | 0.7% | 1.9% | 36.3% | 0.1% | 3.3% | 1.8% | 0.6% | 1.1% | 0.0% | 5.9% |
|  | Coxiellaceae | 6.7% | 0.1% | 3.2% | 0.0% | 0.0% | 0.2% | 0.6% | 0.2% | 0.2% | 1.7% | 0.0% | 0.0% | 0.0% | 87.9% | 0.0% | 1.1% |
|  | Francisellaceae | 0.2% | 0.0% | 0.0% | 0.0% | 0.0% | 0.0% | 0.0% | 0.0% | 0.0% | 0.0% | 0.0% | 0.0% | 3.2% | 0.0% | 0.0% | 0.0% |
|  | Pseudomonadaceae | 1.3% | 0.0% | 0.0% | 0.0% | 0.2% | 0.1% | 0.0% | 0.1% | 0.0% | 0.0% | 0.0% | 0.0% | 18.9% | 0.4% | 0.1% | 0.7% |
|  | Xanthomonadaceae | 1.8% | 0.1% | 0.7% | 0.0% | 0.1% | 0.2% | 0.4% | 9.3% | 0.2% | 0.2% | 0.0% | 12.7% | 0.1% | 0.0% | 0.2% | 2.6% |
|  | Deinococcaceae | 0.5% | 0.9% | 0.0% | 0.0% | 0.0% | 0.0% | 0.0% | 0.0% | 6.9% | 0.0% | 0.0% | 0.0% | 0.0% | 0.0% | 0.0% | 0.0% |
