# Supplementary material for: A study of ticks and tick-borne livestock pathogens in Pakistan
Source: PLoS Negl Trop Dis. 2017 Jun 26;11(6):e0005681. doi: 10.1371/journal.pntd.0005681 (PMC5501686; doi:10.1371/journal.pntd.0005681)
Supplement: S5 Table — (HTML) [file pntd.0005681.s006.html]

Taxa Summaries


|  |  |
| --- | --- |
|  | |
| Taxonomy Summary. Current Level: | |
| View Figure (.pdf)  View Legend (.pdf) |  |
|  |


|  |
| --- |
| View Table (.txt) |

|  |  |  |  |  |  |  |  |  |  |  |  |  |  |  |  |  |  |
| --- | --- | --- | --- | --- | --- | --- | --- | --- | --- | --- | --- | --- | --- | --- | --- | --- | --- |
|  | | Total | Tick.Group1 | Tick.Group2 | Tick.Group3 | Tick.Group4 | Tick.Group5 | Tick.Group6 | Tick.Group7 | Tick.Group8 | Tick.Group9 | Tick.Group10 | Tick.Group11 | Tick.Group12 | Tick.Group13 | Tick.Group14 | Tick.Group15 |
| Legend | Taxonomy | % | % | % | % | % | % | % | % | % | % | % | % | % | % | % | % |
|  | Corynebacterium | 3.0% | 0.2% | 0.3% | 0.0% | 0.0% | 0.0% | 0.0% | 0.0% | 0.9% | 0.0% | 2.4% | 0.1% | 13.3% | 0.0% | 21.9% | 0.8% |
|  | Brachybacterium | 0.0% | 0.0% | 0.0% | 0.0% | 0.0% | 0.0% | 0.0% | 0.0% | 0.0% | 0.0% | 0.0% | 0.0% | 0.0% | 0.0% | 0.0% | 0.2% |
|  | Agrococcus | 0.2% | 0.0% | 3.4% | 0.0% | 0.0% | 0.0% | 0.0% | 0.0% | 0.0% | 0.0% | 0.0% | 0.0% | 0.0% | 0.0% | 0.0% | 0.0% |
|  | Microbispora | 2.2% | 0.0% | 28.7% | 0.0% | 0.0% | 0.0% | 0.0% | 0.0% | 0.0% | 0.0% | 0.0% | 2.1% | 0.0% | 0.0% | 0.0% | 0.1% |
|  | Micrococcus | 0.1% | 0.0% | 1.1% | 0.0% | 0.4% | 0.0% | 0.0% | 0.0% | 0.1% | 0.0% | 0.0% | 0.0% | 0.0% | 0.0% | 0.0% | 0.0% |
|  | Saccharomonospora | 0.3% | 0.0% | 0.0% | 0.0% | 0.0% | 0.0% | 0.0% | 0.0% | 0.0% | 0.0% | 0.0% | 0.0% | 0.0% | 0.0% | 0.0% | 4.5% |
|  | Bacillus | 0.6% | 1.5% | 1.5% | 0.6% | 2.0% | 0.0% | 0.0% | 0.1% | 0.0% | 0.0% | 0.0% | 0.0% | 0.0% | 0.0% | 0.0% | 4.3% |
|  | Paenibacillus | 0.5% | 1.4% | 0.2% | 0.0% | 0.5% | 0.0% | 0.0% | 0.0% | 9.7% | 0.0% | 0.0% | 0.0% | 0.0% | 0.0% | 0.0% | 0.3% |
|  | Planomicrobium | 3.0% | 0.0% | 0.2% | 0.0% | 0.0% | 0.0% | 0.0% | 0.0% | 0.0% | 0.0% | 0.0% | 40.4% | 0.0% | 0.0% | 0.0% | 3.4% |
|  | Staphylococcus | 18.0% | 38.5% | 0.1% | 0.0% | 42.1% | 83.3% | 2.2% | 0.1% | 17.0% | 22.8% | 0.0% | 0.3% | 48.2% | 0.3% | 13.5% | 22.8% |
|  | Enterococcus | 3.4% | 5.7% | 1.0% | 0.4% | 24.8% | 0.1% | 0.0% | 0.2% | 7.0% | 0.2% | 0.6% | 0.3% | 0.0% | 0.0% | 0.0% | 13.9% |
|  | Lactobacillus | 5.2% | 0.0% | 0.0% | 0.2% | 0.0% | 0.0% | 0.0% | 0.3% | 0.3% | 0.0% | 0.0% | 0.3% | 0.3% | 0.3% | 63.9% | 0.2% |
|  | Lactococcus | 5.5% | 0.2% | 0.0% | 0.8% | 0.0% | 0.0% | 0.0% | 0.0% | 0.0% | 65.6% | 0.2% | 0.0% | 0.0% | 0.0% | 0.0% | 0.0% |
|  | Clostridium | 18.0% | 0.0% | 0.0% | 0.8% | 0.0% | 0.1% | 91.1% | 43.0% | 0.3% | 7.8% | 86.7% | 0.0% | 0.2% | 0.0% | 0.1% | 0.3% |
|  | Anaerovorax | 0.8% | 0.0% | 0.0% | 0.0% | 0.0% | 0.0% | 0.0% | 0.0% | 0.0% | 0.0% | 9.8% | 0.0% | 0.0% | 0.0% | 0.0% | 0.0% |
|  | Allobaculum | 0.7% | 0.0% | 0.0% | 0.0% | 0.0% | 0.0% | 0.0% | 10.7% | 0.0% | 0.0% | 0.0% | 0.0% | 0.0% | 0.0% | 0.0% | 0.0% |
|  | Rubellimicrobium | 0.8% | 0.1% | 0.0% | 0.0% | 0.0% | 0.0% | 0.0% | 0.0% | 18.7% | 0.0% | 0.0% | 0.0% | 0.0% | 0.0% | 0.0% | 0.0% |
|  | Rickettsia | 6.1% | 0.0% | 29.7% | 0.0% | 0.0% | 13.6% | 4.4% | 37.7% | 0.0% | 0.0% | 0.0% | 0.0% | 0.0% | 0.0% | 0.0% | 3.5% |
|  | Lautropia | 2.8% | 0.8% | 0.6% | 0.0% | 0.0% | 0.2% | 0.6% | 2.3% | 2.4% | 0.3% | 0.0% | 34.3% | 0.2% | 0.0% | 0.1% | 0.1% |
|  | Ralstonia | 18.3% | 46.1% | 29.7% | 97.1% | 30.0% | 2.6% | 1.0% | 5.3% | 30.1% | 1.5% | 0.3% | 22.2% | 16.1% | 9.8% | 0.4% | 40.0% |
|  | Coxiella | 7.9% | 0.4% | 3.4% | 0.0% | 0.0% | 0.0% | 0.6% | 0.3% | 0.4% | 1.7% | 0.0% | 0.0% | 0.0% | 89.3% | 0.0% | 1.4% |
|  | Francisella | 0.2% | 0.0% | 0.0% | 0.0% | 0.0% | 0.0% | 0.0% | 0.0% | 0.0% | 0.0% | 0.0% | 0.0% | 3.3% | 0.0% | 0.0% | 0.0% |
|  | Pseudomonas | 1.4% | 0.0% | 0.0% | 0.0% | 0.2% | 0.1% | 0.0% | 0.1% | 0.0% | 0.0% | 0.0% | 0.0% | 18.4% | 0.4% | 0.1% | 0.8% |
|  | Lysobacter | 0.2% | 0.0% | 0.0% | 0.0% | 0.0% | 0.0% | 0.0% | 0.0% | 0.0% | 0.0% | 0.0% | 0.0% | 0.0% | 0.0% | 0.0% | 3.2% |
|  | Deinococcus | 0.6% | 5.0% | 0.0% | 0.0% | 0.0% | 0.0% | 0.0% | 0.0% | 13.0% | 0.0% | 0.0% | 0.0% | 0.0% | 0.0% | 0.0% | 0.0% |
